# Vegetation Response to Groundwater Variation in Arid Environments: Visualization of Research Evolution, Synthesis of Response Types, and Estimation of Groundwater Threshold

**DOI:** 10.3390/ijerph16101849

**Published:** 2019-05-24

**Authors:** Feng Huang, Danrong Zhang, Xi Chen

**Affiliations:** 1 College of Hydrology and Water Resources, Hohai University, Nanjing 210098, China; 20030112@hhu.edu.cn; 2 Institute of Surface-Earth System Science, Tianjin University, Tianjin 300072, China; xichen@hhu.edu.cn

**Keywords:** groundwater, vegetation, research evolution, response types, threshold analysis, arid environments

## Abstract

Groundwater depth is an important environmental factor affecting vegetation growth and landscape dynamics in arid environments. This study applied a science mapping approach to visualize the development of groundwater-vegetation-related research, synthesized the vegetation response to changes in groundwater depth, and analyzed the change rate of the response curve to identify the groundwater threshold that is essential to conserve the groundwater-dependent terrestrial ecosystems. These ecosystems emerged as a research hotspot due to climate change, groundwater overexploitation, and the recognition of these ecosystems’ importance for sustainable development. There are two main types of response functions of vegetation to changes in groundwater depth—monotone and bell-shaped functions—among which the monotone function includes linear, curvilinear, and stepwise response. The shape of a response curve is mainly determined by the combined effects of oxygen stress, salinization, and water stress; oxygen stress and salinization dominate in shallow groundwater depth, while water stress dominates in deep groundwater depth. On a non-linear vegetation metric—groundwater depth response curve, the change rate analysis method is effective to identify the breakpoint that can be taken as a candidate threshold of groundwater depth. The results will add insight into the intellectual structure of the groundwater-vegetation interactions and provide practical reference for groundwater resource management, ecological conservation, and sustainable development in arid environments.

## 1. Introduction

Arid environments that can be delineated as semi-arid, arid, and hyper-arid zones cover approximately one-third of the Earth’s land surface [1]. Plant diversity in arid environments is relatively low compared with most other biomes, and vegetation cover is generally sparse, but may be enhanced in the areas where groundwater is available [2]. Terrestrial ecosystems that rely on sub-surface presence of groundwater are kinds of groundwater-dependent ecosystems (GDEs), which are threatened globally because of the unsustainable extraction of groundwater resources [3]. Regions with arid environments are home to over 38% of the world’s population; withdrawals of groundwater for increasing population numbers, along with expanding agricultural and domestic use, cause groundwater decline and severely limit the water availability for groundwater-dependent terrestrial ecosystems (GDTEs) [4]. This results in loss of biodiversity, ecological degradation, land desertification, and environmental deterioration, which in turn negatively affect the social and economic development [5]. On the other hand, vegetation plays an important role in the interactions between groundwater and surface water systems because of its direct and indirect influence on recharge; the long-term pattern of net groundwater discharge is dependent on the following parameters: vegetation distribution, elevation, soil type, and river geometry [6]. In a water cycle, the interactions between vegetation and groundwater mainly occur at two related stages: (a) interference in the processes via which precipitation reaches groundwater reservoir, and (b) extraction of groundwater either through deep roots or by being situated in groundwater discharge areas [7].

In research around the world, attention is paid to sustainable development in arid environments, concerning water allocation between natural and human systems [8]. Therefore, it is essential to understand the interactions between groundwater and vegetation. The interactions were previously reviewed with respect to the following objectives: the relationship between plant communities and changes in groundwater depth [7], ecological water requirement [9], conceptual models illuminating the relationship between vegetation dynamics and groundwater fluctuations [10], relationship between groundwater depth and land desertification and soil salinization [11], impacts of land-use and land-cover changes on recharge rates, evapotranspiration, and water resources [12], application of isotopes to arid-zone hydrology [13], ecohydrological groundwater indicators [14], conservation and restoration of GDEs [15], the impact of climate changes [16], and environmental groundwater depth for the GDTEs [17]. Although much attention was given to the groundwater–vegetation interactions in arid environments, new research developments in the 21st century are rarely reviewed systematically. Moreover, to the best of our knowledge, no previous study visualized information about the development of this field.

GDTEs require the input of groundwater to maintain their current composition and function [18]. The relationship between vegetation and groundwater depth was analyzed previously taking groundwater depth as a driving factor of landscape vegetation patterns; remarkable progress was made in ecohydrological research in arid environments, particularly the influence of hydrologic conditions on ecological processes [19]. Numerous worldwide case studies that were investigated in South Africa, the United States, Australia, and northwest China, for example, concluded that changes in groundwater depth affect the groundwater availability for vegetation and induce changes in vegetation physiology, structure, and community dynamics [7,10,15,20]. Eamus et al. pointed out that the responses were examined extensively at leaf, tree, canopy, population, and community scales, and the response functions for individual vegetation traits were readily apparent [21]. However, as far as we know, synthesis and classification of these apparent response modes is still lacking.

For policy-makers, the information on how vegetation responds to variations in groundwater depth is critical for predicting vegetation growth and will lead to the identification of a groundwater threshold, which indicates the limits of reduction in water-source availability and can be used as an effective parameter for environmental management and protection [22]. When a relationship curve of a vegetation metric as a function of groundwater depth is provided, it is difficult to pinpoint the exact breakpoint with precision so as to identify the groundwater threshold [21]. In some case studies, the groundwater threshold was estimated by observing the response curve, and the results were significantly reliant on researchers’ experience and expertise [23,24]. To eliminate the impact of subjectivity on the results, an objective methodology is necessary to detect the groundwater threshold on the response curve.

Therefore, this study focuses on the groundwater-vegetation interactions in arid environments and aims to (a) provide a visual analysis of research development in the past two decades with particular focus on the latest research; (b) review the literature of case studies and assemble the relationships reported in these disparate studies to determine if any generalized pattern can be extracted between vegetation and groundwater variation, i.e., changes in the depth to groundwater table; and (c) based on the synthesized patterns, discuss a change rate analysis of response curve to detect the groundwater threshold that is essential for the health of the GDTEs. The results will add insight into the intellectual structure of the interactions between groundwater and vegetation in arid environments and indicate the emerging research hotspot of this field. Furthermore, the results will enhance our scientific understanding of vegetation response to groundwater variation in arid environments. The generalized response patterns and the threshold estimation method would be practical in a management context, because they would be helpful for scientifically developing groundwater-related standards that may be applied in all arid environments with GDTEs.

## 2. Research Development of Groundwater-Vegetation Interactions in Arid Environments

### 2.1. Data Acquisition and Visualization Approach

**Figure 1:**
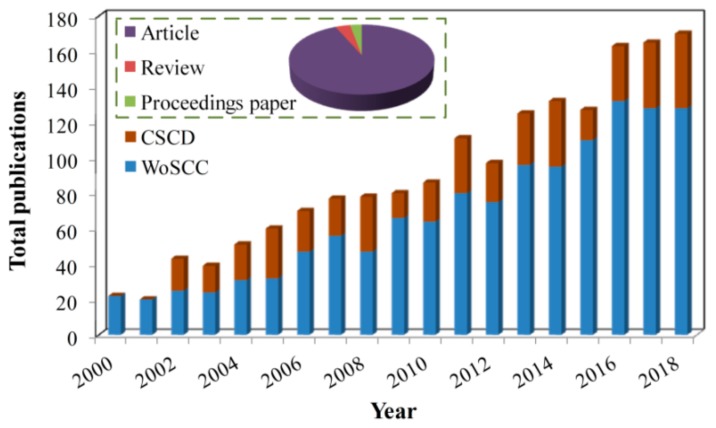
The output performance of research on groundwater–vegetation interactions in arid environments from 2000–2018.

The paper retrieval sources for the analysis were (a) the Web of Science Core Collection (WoSCC) Science Citation Index Expanded (SCI-E), and (b) the Chinese Science Citation Database (CSCD). The search strategies were as follows: TS = (“groundwater*” OR “ground water*” OR “underground water*”) AND TS = (“vegetation*” OR “plant*” OR “grass*” OR “bush*” OR “shrub*” OR “tree*” OR “NDVI*”) AND TS = (“arid*” OR “semiarid*” OR “semi-arid*”), where TS indicates topic search. A series of search terms mentioned above, e.g., “groundwater*”, “vegetation*”, and “arid*”, were scoured in publications containing these words in their topics, including titles, abstracts, and/or keyword lists. The asterisks after the terms are wildcards, which represent any group of characters, including no character. For example, the term “groundwater*” represents both “groundwater” and “groundwater-dependent”. The search results were restricted by document type: article, review, or proceedings. The time span was set as 2000–2018. The search strategies retrieved the vast majority of publications that were related to groundwater and vegetation in arid environments (Figure 1). Generally, there was a rapid growth in the number of publications, implying that more attention is being paid to the scientific and practical problems associated with groundwater and vegetation in arid environments.

The visual analysis was conducted by applying a new version of CiteSpace (5.3.R11, 64-bit) software. This software was proposed by Dr. Chaomei Chen, who systematically introduced the principles and functions of this software in his publications [25]. To perform analysis, the parameters “link retaining factor” and “look back years” were set as −1 and 10, respectively. The time slicing was set as five years per slice, resulting in four sub-periods: 2000–2004, 2005–2009, 2010–2014, and 2015–2018. The term sources used “title”, “abstract”, and “author keywords”. The node type was set as “reference”. The parameter of selection criteria “top N%” was set as “top 5%”. The other parameters were set as default values.

### 2.2. Visualization Results of Research Evolution

**Figure 2:**
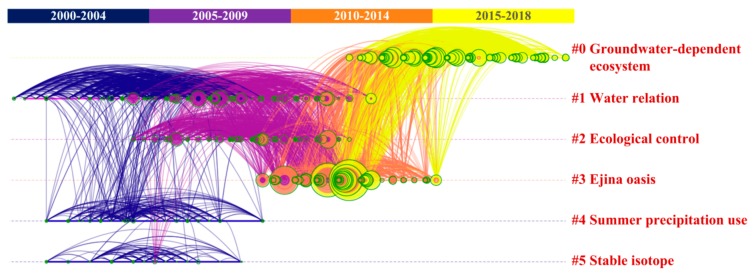
A timeline visualization of the main clusters in the field of groundwater–vegetation interactions in arid environments. Notes: the clusters are arranged vertically in descending order of their sizes; a node represents a reference, and its size is proportional to the cited frequency; the connecting lines between the nodes indicate co-citations.

The document co-citation analysis function in the CiteSpace software constructs networks of cited references, and the synthesized network is further divided into co-citation clusters of references [25]. Figure 2 visualizes a timeline map of clusters in the field of groundwater–vegetation interactions in arid environments. The network had a mean silhouette value of 0.70 and modularity Q of 0.53, indicating that the cluster results were convincing [26]. The silhouette value estimates the uncertainty that is involved in identifying the nature of a cluster, and the modularity Q measures the extent to which a network can be divided into independent clusters [27]. The detailed definitions and formulas of these parameters can be referred to from the published literature, and their results were calculated by the CiteSpace software automatically [28,29]. In Figure 2, each cluster is displayed from left to right along horizontal timelines. In the vertical direction, the largest cluster is displayed at the top of the view, followed by a smaller one. The colored curves represented co-citation links that were added in the year of the corresponding color [25].

Stable isotope and summer precipitation use were two main research domains from 2000–2004. The cluster “#5 stable isotope” was associated with methodologies. Some notable successes in the application of stable isotope included understanding the importance of episodicity and of large flood events to recharge, and delineating sources of water to vegetation [13]. By comparing the hydrogen or oxygen stable isotopic compositions of plant xylem water to those of potential contributive water sources, e.g., water from different soil layers, groundwater, water from recent precipitation, or water from a nearby stream, the researchers can determine the relative contributions of these water sources to root water uptake [30]. The cluster “#4 summer precipitation use” was relevant to water sources of vegetation. For instance, in the arid southwest of North America, hydrogen and oxygen stable isotope ratios of tree xylem water were compared to that of precipitation, groundwater, and deep and shallow soil water to distinguish among possible tree water sources [31]. The cluster “#1 water relation” sustained a period of 2000–2009 and was associated with the vegetation adaptation to arid environment. The research on water relationships of vegetation attempted to explain how the water availability affects the physiological processes that determine the quantity and quality of vegetation growth [32]. From 2005–2009, the cluster “#2 ecological control” emerged as a research domain that concerned the impact of vegetation on water cycle. For example, in the Mojave Desert, the United States of America (USA), the rapid increase in vegetation productivity in response to elevated winter precipitation reduced soil water storage to half of that in a non-vegetated area, thereby precluding deep drainage below the root zone that would otherwise result in groundwater recharge [33].

The cluster “#3 Ejina oasis” represented an important study area from 2010–2014, which is located in the Heihe River basin, northwest China. It was linked with the clusters #0, #1, and #2, indicating that the related scientific issues were studied in the Heihe River basin. Chinese scholars carried out a series of key scientific and technological projects, e.g., “Experiment and Demonstration of Water-Ecology-Economy Management in the Heihe River Basin” and “Integrated Management of Water Resources in the Heihe River Basin” [19]. The cluster “0# groundwater-dependent ecosystem” was the largest cluster and a newly emerged one in recent years. The subsequent analysis and discussion focuses particularly on this research hotspot.

### 2.3. Discussion on the Research Hotspot: GDTEs

The GDEs that are important elements of biodiversity provide valuable goods and services to human society [34]. They can be located in marine, coastal, riparian, in-stream, terrestrial, and in cave and aquifer environments, among which the GDTEs are mostly pertinent with the groundwater-vegetation interactions in arid environments [3]. The research on GDTEs can be traced back to the concept of phreatophyte that was proposed in the 1920s. In the first half of the 20th century, the ecologists and hydrologists mainly studied plants as indicators for groundwater environment, while, in the second half of the 20th century, the ecologists mainly focused on the habitat demands of the phreatophyte [35]. In the 21st century, the GDTEs received more attention because of climate change, e.g., rising air temperature and prolonged drought, and overexploitation of groundwater resources [36]. To achieve sustainable development, the protection and management of GDTEs was incorporated into several water management policy initiatives worldwide, including jurisdictions within Australia, the European Union, South Africa, and the United States [37].

From the perspective of the GDTEs, the research on groundwater–vegetation interactions concerned the following two ecohydrological issues: (a) how much water is taken up by the GDTEs and the impact of transpiration on groundwater fluctuations; and (b) how do the GDTEs respond to groundwater variations, especially groundwater decline due to excessive groundwater extraction [21,35]. In view of the worldwide case studies that attempted to address the second issue, is it possible to categorize the relationships between vegetation and groundwater depth, and how many categories are there? Although the relationships between vegetation and groundwater depth are most likely case-specific due to localized differences in species composition, and climatic and hydrogeological conditions, some generalized information can be synthesized from disparate case studies, and the results may be helpful for policy-makers in groundwater management and environmental protection.

## 3. A Synthesis of Vegetation-Groundwater Response Curves in Arid Environments

### 3.1. Classification Method of Response Functions

The case studies were selected from peer-reviewed publications available through the indexed bibliographic databases: the WoSCC and the CSCD. The application of satellite remote-sensing techniques to monitor vegetation changes enables accurate evaluation of vegetation distribution and dynamics at a large scale; the normalized difference vegetation index (NDVI) that acts as a proxy for the density and photosynthetic capacity of vegetation is strongly correlated with vegetation coverage [38]. When synthesizing the response functions of vegetation to groundwater variation, vegetation metrics included NDVI and its deduced indicators, e.g., vegetation coverage. Furthermore, field-based vegetation coverage was also taken into consideration. The response types were generalized by observing and comparing the relationship curves between a vegetation metric and groundwater depth from disparate case studies. If the relationship curves had a similar tendency, they were classified into the same type.

### 3.2. Classification Results of Vegetation-Groundwater Response Curves

There are two types of vegetation responses to groundwater variation in arid environments: monotone and bell-shaped functions. In the monotone function, vegetation metrics decrease or increase monotonously with declining groundwater table, while, in the bell-shaped function, vegetation metrics switch in increasing and decreasing changes with declining groundwater table and reach an extreme value with a proper groundwater depth. In both functions, when groundwater table drops below a threshold, vegetation is unable to take up groundwater and becomes no longer insensitive to groundwater decline. The monotone response curves of vegetation metric and groundwater depth can be further divided into three types: (a) linear response curve, (b) curvilinear response curve, and (c) stepwise response curve. For example, a linear response can refer to Zhang et al., 2018 [23], a curvilinear response can refer to Wang et al., 2015 [24], a stepwise response can refer to Ma et al., 2009 [39], and a bell-shaped response can refer to Jin et al., 2014 [40].

A negative linear relationship between plant community coverage and groundwater depth was reported in a typical desert riparian ecosystem near the county of Ejin, located at the lower reaches of the Heihe River in an arid area of northwest China [23]. The study region features a typical continental arid climate dominated by warm humid summers and cold dry winters. The multi-year mean precipitation is approximately 37 mm, more than 75% of which falls in July–August, and pan evaporation is about 100 times greater than the precipitation [41]. Generally, vegetation communities changed from the desert riparian forest vegetation of *Tamarix ramosissima* to the typical desert plant community of *Reaumuria songarica* with increasing distance from the river. Community coverage generally decreased with increasing groundwater depth and could be described by a linear equation [23]. The aboveground biomass, community height, and leaf area index were highest for the shallowest groundwater depth due to the contribution of *T. ramosissima* shrub. This species of shrub could survive by extending its roots to a low-salinity alkali underground region, where it is mainly impacted by groundwater depth instead of a high-salinity alkali region [42]. The researchers observed that the desert riparian vegetation was mainly distributed around the 3-m zone of groundwater depth and suggested a lower threshold of 3 m to support the restoration of desert riparian forest [23]. The linear response to changes in groundwater depth was also reported for species richness and leaf area index [23].

A curvilinear decay response of NDVI to groundwater variation was investigated in the Yuka-Daqaidam basin of northwest China [24]. The study area is located in the central basin of a diluvial fan, where groundwater depth decreases gradually and, eventually, a spring emerges. The multi-year mean precipitation is approximately 83 mm, and the evaporation is 2020 mm, which is about 24.3 times greater than the precipitation. When groundwater depth increases from shallow to deep, vegetation types transform following a sequence of hygrophilous vegetation, halophytic vegetation, and xerophytic vegetation. Based on the scatter plot of NDVI and groundwater depth, the researchers identified a lower threshold of 3 m to prevent ecological degradation and land desertification [24]. The scatter plot can be fitted significantly with an exponential function. The curvilinear response of vegetation to groundwater variation was also reported in some other case studies in which the vegetation metrics included herb coverage [43,44,45,46], wood coverage [47], community coverage [48,49,50,51], and tree-ring width indexes [52].

Long-term changes of *Tamarix* vegetation and the driving factors were investigated in the Minqin Oasis, located between the Badain Jaran Desert and Tengger Desert of northwest China [39]. The study area has a multi-year mean precipitation of 116 mm and evaporation of 2644 mm, which is 24 times greater than the precipitation. During the past 50 years, the groundwater level declined widely and continually in the Minqin oasis–desert ecotone, resulting in the dominant plant species changing from mesophytes to xerophytes and, finally, to super-xerophytes. With the increase of groundwater depth, the coverage of *Tamarix* bushes reduced continually, especially when the groundwater depth reached 10–14 m [39]. A sigmoidal function is significantly fitted with the scatter plot that describes such a stepwise negative response. The stepwise positive response of vegetation to groundwater variation was found when studying the canopy dieback as a function of groundwater depth for *Populus fremontii*, *Salix gooddingii*, and *Tamarix chinensis* [53].

A unimodal response of NDVI to groundwater variation was reported in the Dulan area of Qaidam River basin, located in northwest China [40]. The Dulan area has annual precipitation of 25–300 mm and evaporation of 1000–2000 mm. The dominant species of vegetation are *Artemisia desertorum*, *Phragmites*, *Tamarix ramosissima* Ledeb., and *Calligonum arborescens* Litv. When the depth to groundwater table was too shallow or deep, vegetation growth was restrained by salinization, anoxia, or water stress, and a suitable groundwater depth would be beneficial for vegetation glory. Taking an NDVI of 0.08 as a pre-set environmental conservation target, the thresholds of groundwater depth were linearly interpolated based on the scatter plot and the result was 0.7–3.5 m [40]. The scatter plot can be significantly fitted by a Gaussian function. The bell-shaped response of vegetation to groundwater drawdown was also reported in case studies using the following vegetation metrics: wood coverage [45], community coverage [54], NDVI of grassland/scrubland/woodland [55], community NDVI [56,57,58], occurrence frequency [49,59,60,61], and species diversity index [62].

### 3.3. Discussion on Vegetation Response to Groundwater Variation

#### 3.3.1. Physical Causes of Response Types

Hydrology controls the composition of natural vegetation and associated fauna of a site through the water and solute budgets and through the conditions imposed upon the local nutrient cycling [8]. In arid environments, a number of confounding factors may modify the response of vegetation to groundwater variation. The relationship between vegetation and groundwater depth is complicated because it depends a lot on site conditions that mainly include climate, topography, soil, vegetation species, drought and anoxic stress tolerance, changes in the size and distribution of the active root system, associated changes in the water uptake capacity, timing and rate of changes in groundwater regime, herbivory, and disease [10,63,64]. Hydraulic lift is another important factor affecting the relationship between vegetation and groundwater depth. In arid environments, herb plants with shallow root systems contribute greatly to community coverage and species diversity, which may decline due to the disappearance of herb plants with groundwater drawdown [65]. However, shallow-rooted herb species may benefit from the hydraulic redistribution and exist under the tree and shrub layers, because phreatophytic species of trees and shrubs can play a water supplier role for herbs by lifting or transporting water from groundwater [66,67].

Because of multiple impacting factors that are site-specific, the type of vegetation-groundwater variation response curve for a study area may be different with that for another study area; even if the types of response curves are the same, the parameters of the response functions may be different. The main difference between the monotone and bell-shaped response curves is the vegetation response to shallow groundwater depth. For a study area, if native plants can adapt to waterlog and salinization, e.g., if hygrophytes and halophytes exist, the response curve of vegetation to groundwater variation tends to be monotone; otherwise, the response curve is likely to be a bell-shaped one. When groundwater depth varies from shallow to deep, the stress of anoxia and salinization decreases gradually while the stress of water availability increases gradually. The adaption and tolerance of vegetation to these three factors determines the response type of vegetation to groundwater variation.

#### 3.3.2. Integrated Ecosystem-Scale Response

Groundwater plays an important part in sustaining ecosystems and human society, particularly in arid regions where surface water resources are scarce [68]. Policy-makers and managers of water resources and environmental protection commonly ask how much water can be taken from the aquifer while still maintaining a low level of risk to the GDTEs; this requires quantified information on the relationship between the health of the GDTEs and groundwater depth [69]. However, there are uncertainties in the response functions of vegetation to groundwater variation because vegetation characteristics are highly heterogeneous and depend on a multitude of biotic and abiotic factors. In addition, groundwater depth is also highly heterogeneous at global and regional scales due to complex landscapes and aquifers [70]. Therefore, sufficient monitored data, e.g., remotely sensed data and field data, and rigorous statistical methodologies are necessary to delineate the response curves.

Since the responses of vegetation to changes in groundwater depth were investigated extensively at leaf, tree, canopy, and population scales, and the response functions for individual traits are readily apparent, more efforts are still required to examine multiple traits across multiple scales and provide an integrated ecosystem-scale response function to groundwater variation [21]. Theoretically, the integrated ecosystem-scale response to changes in groundwater availability is hypothesized to be a linear, a curvilinear, or a step function [3]. An ecosystem response can be considered as a comprehensive response of individual vegetation traits. There are linear, curvilinear, and stepwise response functions between vegetation metrics and groundwater depth; hence, the hypothesis of ecosystem response is reasonable. However, the hypothesis is incomplete because there is also a bell-shaped response function. If oxygen stress and salinization caused by shallow groundwater depth and water stress caused by deep groundwater depth restrain the health of a GDTE, the ecosystem may show a bell-shaped response to groundwater drawdown. For example, in a desert riparian forest at the lower reaches of the Trim River, northwest China, the most appropriate groundwater depth for the optimal composition and structure of plant communities is 2–6 m; shallower and deeper groundwater depth will cause ecosystem degradation, e.g., reduced vegetation density and coverage [45]. Practically, more case studies are still required to verify the rationality of this hypothesis.

## 4. Estimation of Groundwater Threshold for Conserving the GDTEs

### 4.1. Methodology for Estimating Groundwater Threshold

After the response modes were categorized, the sensitivity of the vegetation metric to changes in groundwater depth was analyzed to indicate the groundwater threshold. An ecological threshold is the point where small changes in an environmental driver produce large responses in the ecosystem [71]. In arid environments, groundwater is an essential water source for vegetation, and the depth to groundwater table is a vital factor affecting the community structure and dynamics of the GDTEs. Under natural conditions, vegetation grows well if the capillary fringe is near the root zone [11]. For each vegetation individual of different species or different ages, the root depths and their tolerance to environmental stresses caused by too shallow or too deep groundwater depth are different. When the environmental stresses restrain the vegetation growth, e.g., when the capillary fringe is far from the root zone and the groundwater is unavailable, vegetation may become no longer insensitive to groundwater decline. Therefore, on a response curve of vegetation to groundwater variation, the groundwater depth of a breakpoint that differentiates sensitive and insensitive responses can be considered as a candidate groundwater threshold.

To eliminate the potential impact of relative scaling of vegetation metric and groundwater depth, dimensionless values were used to fit the function of response curve. Both vegetation metric and groundwater depth were normalized and expressed as a proportion of their maximum values, i.e., normalized vegetation metric (NVM) and normalized groundwater depth (NGD). A first-order derivative of the fitted response function was calculated, and the change rate of the NVM-NGD curve was analyzed. At the point of the response curve where the absolute value of slope is equal to 1, a small variation in NGD will produce the same variation in NVM. A larger absolute value of slope indicates more prominent sensitivity of vegetation to groundwater depth, and vice versa. If the absolute value of slope is larger than 1, a small decrease in NGD will produce a large increase or decrease in NVM, indicating that NVM is sensitive to NGD. Conversely, if the absolute value of slope is smaller than 1, the sensitivity of NVM to NGD is lower and changes in NGD will not produce prominent changes in NVM. Hence, the breakpoint on the NVM-NGD curve with the absolute value of slope equal to 1 was suggested to be a candidate threshold of groundwater depth.

### 4.2. Results of Groundwater Threshold

**Figure 3:**
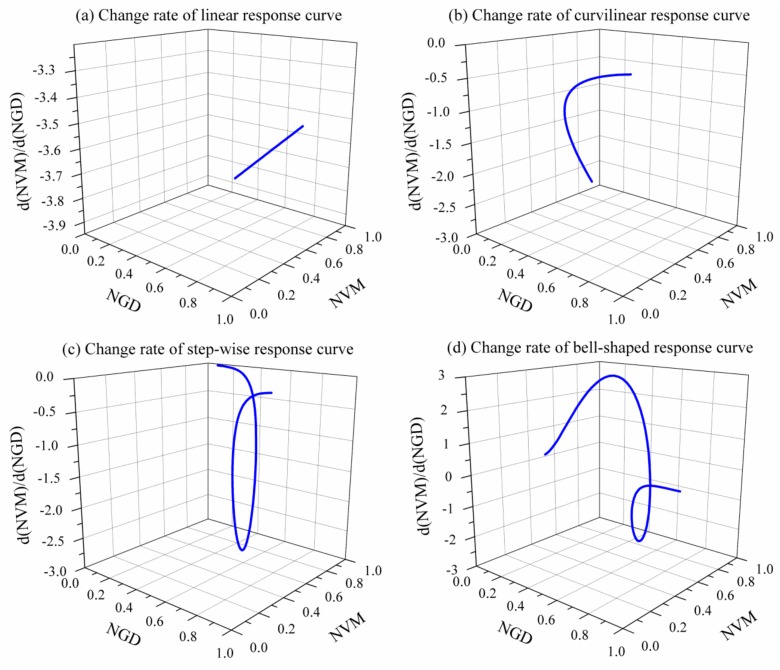
Change rate of normalized vegetation metric (NVM) and normalized groundwater depth (NGD) response curve, (**a**) Change rate of linear response curve, (**b**) Change rate of curvilinear response curve, (**c**) change rate of step-wise response curve, (**d**) change rate of bell-shaped response curve.

The data of vegetation metrics and groundwater depth from exemplary case studies [23,24,39,40] were normalized from zero to one by expressing the maximum trait value as 1 and all other values for that trait as a fraction of the maximum. The normalized scatter plots were fitted by linear, exponential, sigmoidal, and Gaussian functions, based on which the sensitivity of vegetation metrics to changes in groundwater depth were further analyzed (Figure 3). Figure 3a displays a straight line that indicates a constant change rate of a linear response curve, and the other three sub-figures display curves which indicate varying change rates. Specifically, in Figure 3b, the change rate of a curvilinear response curve increases gradually with the increasing NGD and decreasing NVM. In Figure 3c, the change rate of a stepwise response curve decreases firstly and then turns to increase when the NGD increases and the NVM decreases. In Figure 3d, when the NGD and NVM both increase, the change rate of a bell-shaped response curve increases firstly and then turns to decrease; otherwise, when the NGD increases but the NVM decreases, the change rate decreases firstly and then turns to increase.

The slope of the linear response curve is a constant value that indicates a stable sensitivity of vegetation metrics to groundwater fluctuations. Therefore, the change rate analysis is not applicable for the linear response to identify the candidate threshold of groundwater depth. The fitted linear response curve has an intersection with the *X*-axis, and the point of intersection can be considered as a candidate breakpoint. When groundwater depth increases to approximately 3.2 m, the community coverage reduces to 0 [23], suggesting a risk of vegetation dieback and land degradation. Hence, 3.2 m of groundwater depth can be considered as a threshold for groundwater resource management.

For the exponential decay response [24], when groundwater depth varies from shallow to deep, the reduction rate of NDVI changes from large to small. The change rate analysis method detected a candidate breakpoint with a groundwater depth of 2.5 m and NDVI of 0.2. When groundwater depth is shallower than 2.5 m, NDVI is sensitive to groundwater drawdown and the negative response is dramatic. However, when groundwater table declines and the depth is larger than 2.5 m, the sensitivity of NDVI to groundwater drawdown reduces, and the change rate of response curve decreases gradually approaching 0. The reduced sensitivity, especially when the change rate reaches near 0, indicates that the accessibility of a permanent groundwater source decreases and there is potential water stress. Therefore, a threshold of groundwater depth with 2.5 m can be considered as a reference for groundwater resource management. 

The change rate analysis identified two breakpoints for the sigmoidal response curve [39]. The candidate thresholds of groundwater depth are 9.4 m and 14.8 m, corresponding to coverages of *Tamarix* bushes of 22% and 9%, respectively. When groundwater depth is shallower than 9.4 m, *Tamarix* bushes can take up groundwater effectively, resulting in small negative changes responding to groundwater drawdown. When groundwater depth increases from 9.4 m to 14.8 m, the coverage of *Tamarix* bushes decreases dramatically, indicating a sensitive response of *Tamarix* bushes to groundwater drawdown. When groundwater depth increases to deeper than 14.8 m, *Tamarix* bushes have very limited access to groundwater and become no longer insensitive to groundwater decline. Therefore, 14.8 m of groundwater depth can be considered as a critical threshold, below which groundwater cannot be an effective water source for *Tamarix* bushes. Furthermore, 9.4 m of groundwater depth corresponding to a coverage of *Tamarix* bushes of 22% can be considered as a desirable environmental management target that benefits both ecological protection and groundwater exploitation.

For the bell-shaped response [40], four candidate thresholds of groundwater depth were detected using the change rate analysis method: 0.7 m, 1.6 m, 1.9 m, and 2.8 m. When groundwater depth is shallower than 0.7 m, evaporation is intensified and the salt dissolved in the groundwater moves to the topsoil, resulting in soil salinization [11]. Moreover, the soil aeration may be affected and the root respiration may be restrained. Hence, vegetation growth may be restricted by salinization or oxygen stress, resulting in an insensitive response of NDVI to changes in groundwater depth. Below the depth-to-groundwater threshold of 0.7 m, the stress of salinization and anoxia reduces with the increased groundwater depth, and vegetation can take up groundwater effectively. In the range 0.7–1.6 m of groundwater depth, NDVI increases significantly with groundwater variation. The range 1.6–1.9 m of groundwater depth satisfies the vegetation growth not under the stress of salinization, anoxia, or water availability, resulting in a relatively high value of NDVI. In this range, NDVI is insensitive to changes in groundwater depth and fluctuates slightly with groundwater variation. However, when groundwater depth increases deeper than 1.9 m, groundwater availability reduces with the increased groundwater depth and water stress may restrain vegetation growth gradually. In the range 1.9–2.8 m of groundwater depth, NDVI is sensitive to changes in groundwater depth and decreases dramatically with groundwater drawdown. When groundwater depth increases to deeper than 2.8 m, vegetation has a very limited access to groundwater and becomes no longer insensitive to groundwater decline. Therefore, 0.7 m and 2.8 m can be considered as upper and lower thresholds of groundwater depth, respectively. The range 1.6–1.9 m of groundwater depth is suitable for vegetation growth and can be considered as a desirable environmental management target.

### 4.3. Discussion on Estimation of Groundwater Threshold

Given a response curve of vegetation to groundwater variation, it is important to detect a threshold of groundwater depth to inform water resource management and environmental conservation. In the practice of riverine ecosystem protection, the change rate analysis was successfully applied to estimate environmental flows and balance the trade-off between habitat conservation and water resource utilization. Given a wetted perimeter-discharge curve, it is not possible to select the breakpoint reliably by eye; one reliable method of determining the breakpoint is to select the point on the curve where the slope is equal to a nominated value, usually set as 1 [72]. However, in the practice of the GDTE protection, a well-recognized statistical method to detect the breakpoint on a vegetation metric-groundwater depth curve is still lacking, and such a change rate analysis has great potential because of its effectiveness.

For a linear vegetation metric-groundwater depth curve, the change rate analysis is not applicable because the slope of the curve is constant; this method is applicable for a non-linear response curve. To make the changes in vegetation metric and groundwater depth comparable, it is necessary to normalize the axes of vegetation metric and groundwater depth to cover the same range; this can be done by expressing each vegetation metric and groundwater depth value as a proportion of their respective measured maximum values. At the point where the absolute value of curve slope is equal to 1, a small change in groundwater depth (as a percentage of the maximum value considered) will produce the same change in vegetation metric (as a percentage of the maximum value considered). Otherwise, a small change in groundwater depth will produce a large or smaller change in vegetation metric, indicating the changes in sensitivity of vegetation to groundwater variation. Therefore, theoretically, it is reasonable to take the value of groundwater depth corresponding to the detected breakpoint as a candidate threshold. In terms of practice, the application of the change rate analysis (Figure 3) further proved its effectiveness and reasonability. Additionally, more case studies are required to verify the applicability and reliability of the change rate analysis.

As discussed in Section 3.3.1, because the factors that affect vegetation response to groundwater variation are site-specific, the vegetation–groundwater variation response curves may be also site-specific, e.g., the response types may be different, or the parameters of the response functions may be different. Therefore, the change rate analysis will result in site-specific groundwater thresholds for different case studies. For example, the groundwater thresholds for preventing water stress ranged from 2.5 m to 14.8 m for the four case studies [23,24,39,40].

## 5. Conclusions

The achievement of this study includes quantitatively visualizing information about the development of groundwater-vegetation-related research over the past two decades, the types of vegetation metric-groundwater depth response curves, and a method to identify a groundwater threshold on a response curve. Correspondingly, the conclusions and the implications for groundwater resources management, ecological conservation, and sustainable development in arid environments are as follows:

(a) The research on groundwater–vegetation interactions in arid environments includes multiple areas, e.g., water use strategy of vegetation, the impact of vegetation on water cycle, and vegetation response to groundwater availability. Climate change and anthropogenic activities necessitate a deeper understanding of the roles that groundwater plays in GDTEs, which emerged as a research hotspot. In future research, of particular importance is the elucidation of groundwater’s effect on the sustainability of GDTEs.

(b) Vegetation may respond to groundwater variation in two types of modes, including monotone and bell-shaped functions. The monotone response curves can be further divided into three types: linear, curvilinear, and stepwise. Multiple biotic and abiotic factors affect vegetation response to groundwater variation, resulting in site-specific response types and various parameters of response functions. The combined effects of oxygen stress, salinization, and water stress mainly determine the types of response functions. The response curves provide information for ecological conservation, and they help policy-makers predict vegetation growth in the context of groundwater variation and design strategies to prevent ecological deterioration. 

(c) The change rate analysis method is effective to detect the breakpoint on a vegetation metric–groundwater depth response curve that is curvilinear, stepwise, or bell-shaped. The breakpoint indicates the change in sensitivity of vegetation metric to groundwater variation, and the corresponding groundwater depth can be considered as a candidate threshold. For a linear response curve, the change rate analysis method is not applicable; the groundwater depth corresponding to the intersection of the linear response curve and the *X*-axis can be considered as a candidate threshold. The threshold estimation methodology is practical in groundwater resource management because it objectively assesses the groundwater depth that is critical for ecological conservation and helps estimate how much groundwater can be exploited for socio-economic development.

In summary, this study adds scientific insight into vegetation response to groundwater variation and provides a methodology for estimating groundwater threshold; the primary findings can technically support sustainable development in arid environments. However, there are still some deficiencies. This study synthesized the response functions of vegetation to groundwater variation based on peer-reviewed publications, but unpublished materials, e.g., research reports and academic presentations, were not considered due to data limitation. Therefore, the synthesis of response functions may be incomplete. In addition to linear, curvilinear, stepwise, and bell-shaped response functions, there may be other types of responses reported in the unpublished materials. Because of this limitation, another limitation is that the applicability and effectiveness of the change rate analysis to the unpublished response curves cannot be assessed. Furthermore, more case studies are required to verify the applicability and reliability of the change rate analysis to detect breakpoints on response curves of vegetation to groundwater variation.
